# Tuning Nano-Amorphous Calcium Phosphate Content in Novel Rechargeable Antibacterial Dental Sealant

**DOI:** 10.3390/ma11091544

**Published:** 2018-08-27

**Authors:** Maria Salem Ibrahim, Faisal D. AlQarni, Yousif A. Al-Dulaijan, Michael D. Weir, Thomas W. Oates, Hockin H. K. Xu, Mary Anne S. Melo

**Affiliations:** 1 Program in Dental Biomedical Sciences, University of Maryland School of Dentistry, Baltimore, MD 21201, USA; msyibrahim@gmail.com; 2 Department of Preventive Dental Sciences, College of Dentistry, Imam Abdulrahman bin Faisal University, Dammam 34212, Saudi Arabia; 3 Department of Substitutive Dental Sciences, College of Dentistry, Imam Abdulrahman bin Faisal University, Dammam 34212, Saudi Arabia; falqarni@icloud.com (F.D.A.); yaldulaijan@gmail.com (Y.A.A-D.); 4 Department of Advanced Oral Sciences and Therapeutics, University of Maryland School of Dentistry, Baltimore, MD 21201, USA; MWeir@umaryland.edu (M.D.W.); TOates@umaryland.edu (T.W.O.); HXu@umaryland.edu (H.H.K.X.); 5 Center for Stem Cell Biology & Regenerative Medicine, University of Maryland School of Medicine, Baltimore, MD 21201, USA; 6 Marlene and Stewart Greenebaum Cancer Center, University of Maryland School of Medicine, Baltimore, MD 21201, USA

**Keywords:** dental sealant, resin sealant, calcium phosphate nanoparticles, long-term ion release, remineralization, ion recharge

## Abstract

Dental sealants with antibacterial and remineralizing properties are promising for caries prevention among children and adolescents. The application of nanotechnology and polymer development have enabled nanoparticles of amorphous calcium phosphate (NACP) and dimethylaminohexadecyl methacrylate (DMAHDM) to emerge as anti-caries strategies via resin-based dental materials. Our objectives in this study were to (1) incorporate different mass fractions of NACP into a parental rechargeable and antibacterial sealant; (2) investigate the effects on mechanical performance, and (3) assess how the variations in NACP concentration would affect the calcium (Ca) and phosphate (PO_4_) ion release and re-chargeability over time. NACP were synthesized using a spray-drying technique and incorporated at mass fractions of 0, 10, 20 and 30%. Flexural strength, flexural modulus, and flowability were assessed for mechanical and physical performance. Ca and PO_4_ ion release were measured over 70 days, and three ion recharging cycles were performed for re-chargeability. The impact of the loading percentage of NACP upon the sealant’s performance was evaluated, and the optimized formulation was eventually selected. The experimental sealant at 20% NACP had flexural strength and flexural modulus of 79.5 ± 8.4 MPa and 4.2 ± 0.4 GPa, respectively, while the flexural strength and flexural modulus of a commercial sealant control were 70.7 ± 5.5 MPa (*p* > 0.05) and 3.3 ± 0.5 GPa (*p* < 0.05), respectively. A significant reduction in flow was observed in the experimental sealant at 30% NACP (*p* < 0.05). Increasing the NACP mass fraction increased the ion release. The sealant formulation with NACP at 20% displayed desirable mechanical performance and ideal flow and handling properties, and also showed high levels of long-term Ca and PO_4_ ion release and excellent recharge capabilities. The findings provide fundamental data for the development of a new generation of antibacterial and rechargeable Ca and PO_4_ dental sealants to promote remineralization and inhibit caries.

## 1. Introduction

Dental caries is still a highly prevalent oral disease worldwide despite the various approaches that have been used to prevent it [1,2]. These approaches include fluoride exposure, sugar intake control, brushing and dental sealants [3]. Dental sealants help prevent caries in pits and fissures of primary and permanent teeth, acting as a physical barrier for food accumulation and bacterial growth [4]. Accumulative evidence from epidemiologic findings has shown positive outcomes for caries prevention when the teeth are sealed, in comparison to non-sealed teeth in children and adolescents [4,5]. It was found that sealants on permanent molars may reduce dental caries for up to 24–48 months when compared to that of no sealant application [3,4]. In terms of sealants’ retention, resin-based sealants are the materials with a higher success rate [6]. Even so, findings have shown an increase in dental resin-based sealants failures due to bacterial colonization under the restored fissures, thereby, initiating and progressing the carious lesion beneath the sealant [4].

Caries lesions at the sealed occlusal surfaces were initiated when the balance between the remineralization and demineralization of the tooth structure was adversely affected, and demineralization exceeded the remineralization abilities, resulting in the dissolution of hydroxyapatite crystals [7]. To allow the remineralization process to occur, adequate levels of calcium (Ca) and phosphate (PO_4_) ions must be available [8]. Antibacterial and remineralizing resin-based sealants could be one of the most desirable approaches for management of dental caries in children and adolescents due to the potential of reduced bacteria and provide localized ion release near the tooth surface.

Recently, new fundamental research findings have highlighted the application of nanotechnology and polymer development in dental materials, enabling nanoparticles of amorphous calcium phosphate (NACP) and quaternary ammonium methacrylate such as dimethylaminohexadecyl methacrylate (DMAHDM) to emerge as anti-caries strategies via resin-based materials [9,10]. Amorphous calcium phosphate (Ca_3_[PO_4_]_2_), as a precursor of the final crystalline hydroxyapatite, has been investigated as a suitable remineralizing agent. There is a growing body of evidence suggesting that NACP could enhance the remineralizing capacity due to a greater surface area-to-volume ratio [11,12]. NACP had a relatively high specific surface area of 17.76 m^2^/g, compared to about 0.5 m^2^/g of traditional micron-sized calcium phosphate particles used in dental resins [13,14]. Supported by the high performance of Ca and PO_4_ ion release provided by a nanostructured compound, NACP has led to new possibilities for combating enamel demineralization [15].

Resin-based sealants with the ability to release Ca and PO_4_ ions are expected to suppress the demineralization process and prevent dental caries. However, ion-depletion effect with loss of bioactivity over time (short-term ion release) has been a major drawback for calcium phosphate-containing resins [6,16]. Recently, significant levels of Ca and PO_4_ ions were released from NACP-containing resins that were sustainable over long periods of time and with rechargeable capacity [17,18]. The rechargeable capability of NACP-containing dental materials have opened new horizons and are expected to lead to relevant changes in remineralizing approaches [19]. The repeatable recharge process to re-release Ca and PO_4_ ions can lead to supersaturation into the surrounding microenvironments under acidic attacks, such as enamel areas located within deep occlusal pits and fissures with difficult access to brush. These ions can play a vital role in the precipitation of crystallites. This capability is highly desirable in a resin-based formulation for sealing the occlusal pits and fissures where high rates of demineralization happen and result in almost 50% of all caries in school children [2,4].

Many of the innovative bioactive strategies and technologies require new dental materials with new combinations of properties to meet the basic properties of the conventional polymeric materials [13]. This is true for resin-based formulations that are needed for dental sealant applications. For example, regarding the chemical and physical characteristics, the resin-based sealants must possess a high degree of wettability, and flow and viscosity that allow the penetration between the occlusal fissures and grooves of the teeth [5]. Another important characteristic is the resistance to abrasion and fracture, which would demonstrate the adequate mechanical performance of the material [6]. This is a challenging combination of characteristics when developing new resin-based formulations. Frequently, the incorporation of new agents in the resin-based system decreases the strength or bioactivity.

The present study reports the development of new antibacterial resin-based sealants that include NACP for Ca and PO_4_ ion release and recharge properties. Our objectives were to (1) incorporate different mass fractions of NACP into parental rechargeable antibacterial sealant; (2) investigate the effects on mechanical performance; and (3) assess how the variations in NACP concentration would affect the Ca and PO_4_ ions release and re-chargeability over time. It was hypothesized that adding an increased percentage of NACP would have acceptable mechanical and physical performances, while producing substantial initial ion release and a long-term repeated recharge capability.

## 2. Results

**Figure 1:**
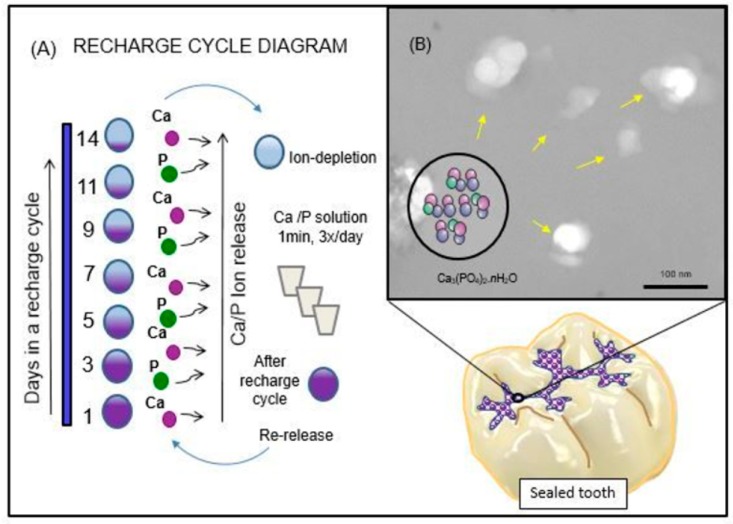
Schematic diagram of the rechargeable nanoparticles of amorphous calcium phosphate (NACP) sealant approach to deal with enamel demineralization around dental sealants: In (**A**), the recharge cycle diagram illustrates the re-release from the exhausted and recharged NACP sealants. Three recharge/re-release cycles were performed, and each re-release was measured for 14 days. The ion re-release increased with increasing the NACP filler level. In (**B**), the TEM image of NACP from the spray-drying technique having sizes of about 100–300 nm.

Illustration of the rechargeable NACP approach to dealing with the dissolution-diffusion process of enamel demineralization around dental sealants is shown schematically in Figure 1. The ion recharge cycle diagram displayed the ion re-release from the exhausted and recharged NACP-containing resin-based sealants in Figure 1A. Three recharge/re-release cycles were performed, and each re-release was measured for 14 days. The ion re-release increased with increasing the NACP filler level. Figure 1B shows the TEM image of NACP synthesized using the spray-drying technique having sizes of about 100–300 nm. The structural model of amorphous calcium phosphate is exemplified in this image, and the potential application for sealing the occlusal surface of the posterior teeth is illustrated.

**Figure 2:**
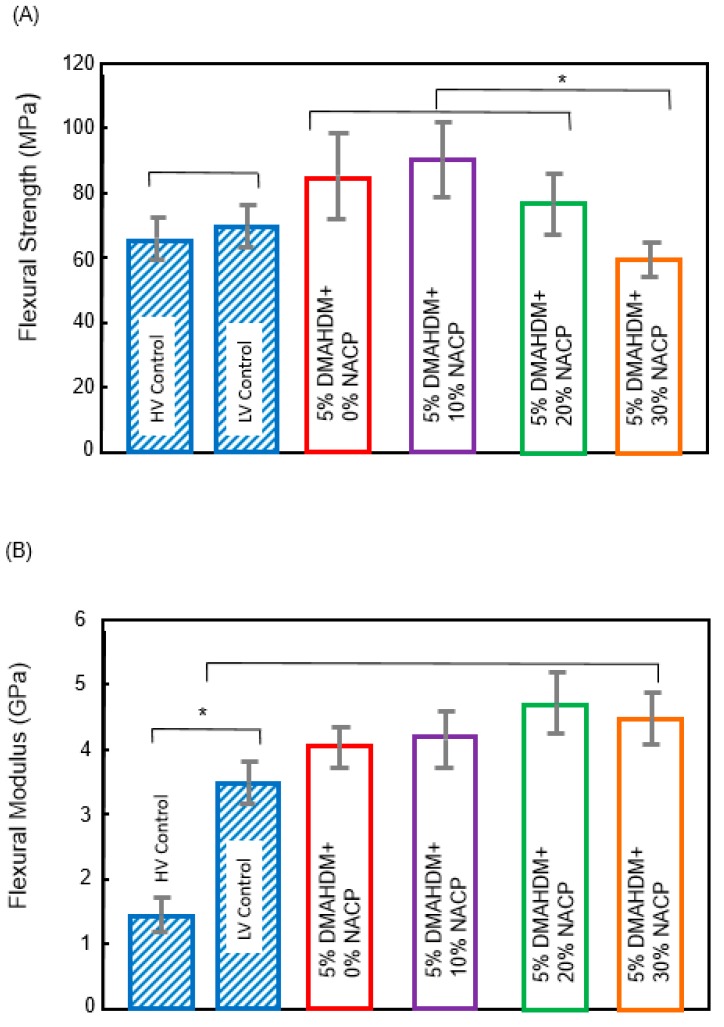
Bar graphs of (**A**) flexural strength and (**B**) flexural modulus (Mean ± SD; *n* = 8) of resin-based sealants. The asterisk means that there was a statistically significant difference between the groups.

Figure 2 describes the flexural strength and modulus of the antibacterial and rechargeable resin-based sealants (Mean ± SD; *n* = 8). In Figure 2A, the flexural strength showed a decreasing trend with increasing NACP percentage, because the glass filler level was decreasing from 50% to 20%. The experimental sealant at 20% NACP had the flexural strength and flexural modulus of 79.5 ± 8.4 MPa and 4.2 ± 0.4 GPa, respectively. The commercial high viscosity sealant control showed flexural strength and flexural modulus of 70.7 ± 5.5 MPa (*p* > 0.05) and 3.3 ± 0.5 GPa (*p* < 0.05). These results demonstrated that the rechargeable antibacterial sealant at 20% mass fraction of NACP had mechanical properties similar to or higher than that of the commercial sealant/flowable composite control. Only the sealant with 30% NACP had a significantly lower strength that those other groups (*p* < 0.05).

**Figure 3:**
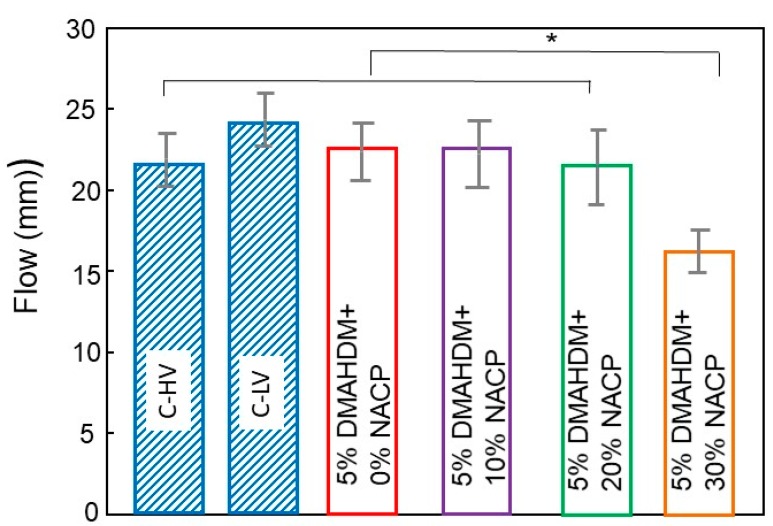
Means ± SD of flow analysis of the resin-based sealant formulations (*n* = 6). The asterisk means that there was a statistically significant difference between the groups.

The flow results for the experimental and control resin-based sealants (Mean ± SD; *n* = 6) are plotted in Figure 3. NACP at a mass fraction of 30% compromised the flow of the sealant (*p* < 0.05). Experimental sealant with a mass fraction of 20% NACP had a flow that was not significantly different from the control (*p* > 0.05).

**Figure 4:**
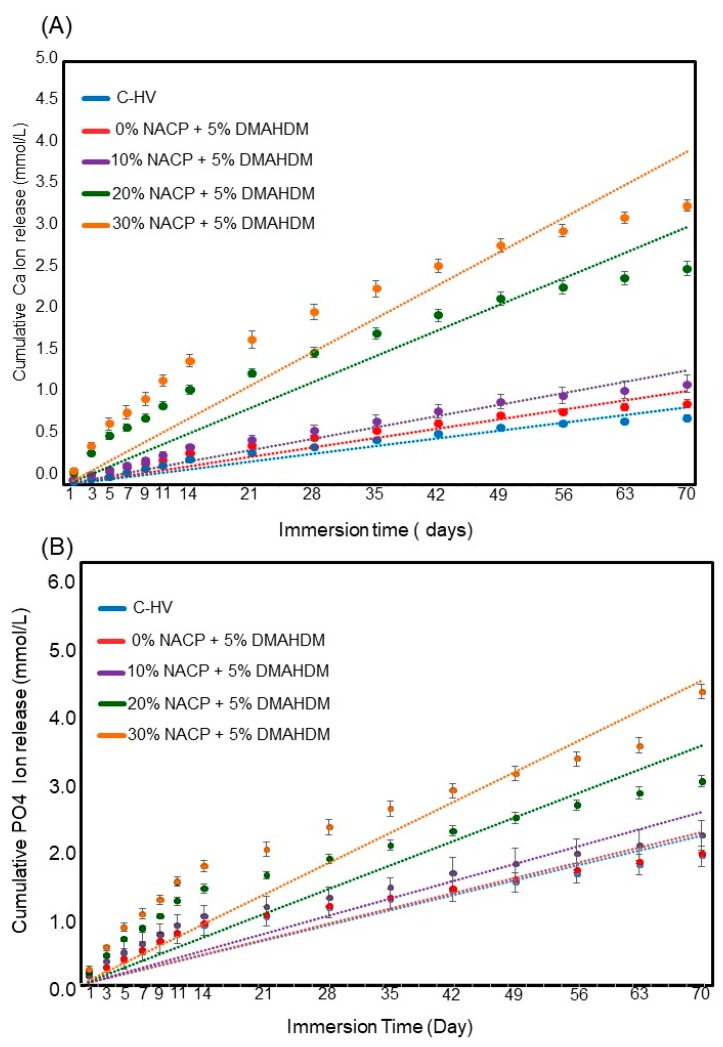
Cumulative initial ions release from sealants (Mean ± SD; *n* = 6). In (**A**), Calcium (Ca) ion and in (**B**) Phosphate ions. The dots for each group show the exact data and the dotted line is its approximate linear trend for each formulation.

Figure 4 plots the Ca and PO_4_ initial ion release over time (Mean ± SD; *n* = 6). After 70 days of ion release, 30% NACP + 5% DMAHDM sealant had higher Ca ion release of 4.70 ± 0.95 mmol/L. This amount was significantly different from 20% NACP + 5% DMAHDM sealant that released 3.64 ± 0.11 mmol/L (*p* < 0.05). The PO_4_ ion release had similar release behavior. The 30% NACP + 5% DMAHDM sealant showed 4.25 ± 0.12 mmol/L of PO_4_ ion release, followed by the 20% NACP + 5% DMAHDM with 3.41 ± 0.10 mmol/L of PO_4_ ion release (*p* < 0.05). During the 70 days, the pH increased from approximately 4.5 to 6.5 for the groups of the formulations containing 20 and 30% NACP. These changes represent the neutralizing capabilities of calcium and phosphate ion release.

**Figure 5:**
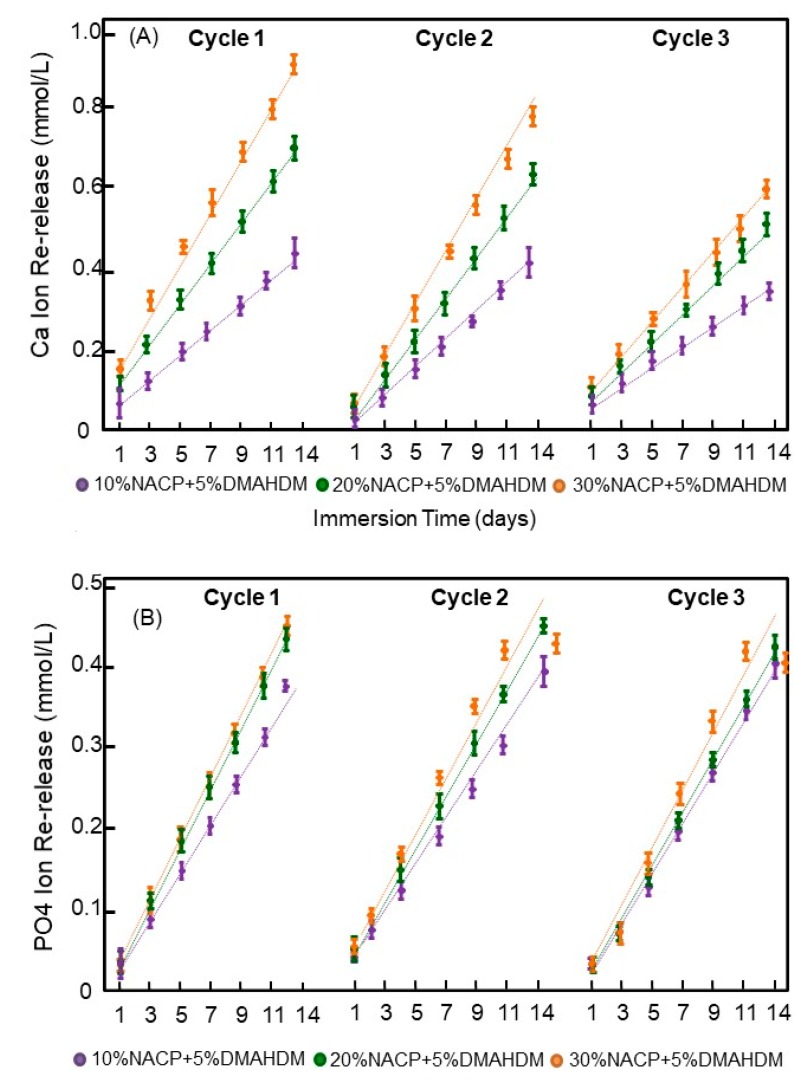
Cumulative ions re-release from the recharged resin dental sealants (Mean ± SD; *n* = 3) after three cycles of ions recharge and re-release. The dots for each group show the exact data and the dotted line is its approximate linear trend for each formulation. In (**A**), Calcium ion and in (**B**) Phosphate ions. There was no decrease in the ion re-release amounts with increasing the number of recharge and re-release cycles.

The results of the three cycles of ion recharge and re-release are presented in Figure 5. The Ca ion re-releases after the first cycle were 0.46 ± 0.03, 0.73 ± 0.03, and 0.94 ± 0.04 for the experimental sealants with 10%, 20% and 30% NACP, respectively (*p* < 0.05 between the three groups). On the other hand, the PO_4_ ion re-release after the first cycle were 1.10 ± 0.01, 1.22 ± 0.02, and 1.25 ± 0.03 for the experimental sealants with 10%, 20% and 30% NACP, respectively (*p* > 0.05 between the 20% and 30% NACP groups). All the experimental sealants showed good Ca and PO_4_ ion recharging abilities in the three cycles tested.

## 3. Discussion

Recent studies have developed rechargeable NACP resin-based materials for long-term Ca and PO_4_ ion release to combat tooth decay [17,19], but the effects of NACP filler level on antibacterial sealant properties had not been reported. Developing a new dental material for sealant applications must meet the basic property requirements of clinicians. Thus, in the present study, an antibacterial and rechargeable Ca and PO_4_ releasing sealant was developed for the first time. The effects of NACP mass fraction on mechanical and physical performance and Ca and PO_4_ ions initial release and re-release were determined to allow the development of ideal formulation with bioavailable ions for potential enamel remineralization of occlusal pits and fissures.

In the case of the enlarged pit and fissure sealing, the mechanical properties of the material become more important since the material can be placed onto areas that encounter mechanical stresses during clenching [6]. In that situation, it appears clearly from the results that flowable resin composites have by far better flexural moduli than the pit and fissure sealants tested. Here, we assessed the flexural strength, flexural modulus and flow of different formulations and compared them to the commercial resin-based sealant and flowable composite that did not contain NACP. The percentage load of the glass filler ranged from 50% in 5% DMAHDM + 0% NACP sealant to 20% in 5% DMAHDM + 30% NACP formulation. Previous studies also revealed that the incorporation of a high content mass fraction of nanoparticles, such as 40% might have a negative impact on material properties [20,21]. Therefore, only three different mass fractions of NACP were used in this study: 5% DMAHDM + 10% NACP, 5% DMAHDM + 20% NACP, and 5% DMAHDM + 30% NACP. However, the results obtained in this study revealed a significant interference of the NACP at the 30% mass fraction with the flow of the experimental sealant. In deep fissures and grooves, the flow of the material is an important characteristic to help seal the total surface [4]. It is expected that the sealant’s flow ability could be compromised when the experimental sealant incorporates the highest amount of NACP. Our findings suggest that the new formulation with 20% NACP did not negatively affect the mechanical and physical properties, while providing substantial Ca and PO_4_ ions to inhibit caries.

Adding different caries-preventive measures and strategies to the sealant may increase its effect on caries prevention [5]. In previous studies, antibacterial resins containing quaternary ammonium methacrylates with an alkyl chain length of 16 (DMAHDM) were synthesized and assessed with a higher antibacterial potency against oral bacteria as an outcome. The mechanism of positively charged quaternary amine disrupting the negatively charged bacterial membranes supported the decrease in bacterial coverage nearly 90% for resin formulations at 5% DMAHDM [17,19]. The long-term antibacterial activity of DMAHDM was demonstrated by Zhang et al. [20] and attributed to the fact that the antibacterial monomer was copolymerized with the resin by forming a covalent bonding with the polymer network. Thus, the development of a resin-based dental sealant that also holds remineralizing properties could be essential to improve the function of the resin-based dental sealant on posterior teeth fissures and grooves for caries prevention.

Regarding recharge ability, this study builds upon the methodology provided by Zhang et al. [21]. Their report confirmed that the recharge of calcium and phosphate ions from resin-based formulations was achievable, while also establishing some of the variables important for ion release and recharge such as chemical composition of resin matrix and number of suitable cycles for recharge of the specimens. For the tested formulations, the 20% NACP sealant showed a release of 3.64 ± 0.11 mmol/L (*p* < 0.05) of Ca ions and 3.41 ± 0.10 mmol/L of PO_4_ ions during the 70-day period. The effect of the NACP percentage loading within the resin-based antibacterial formulation containing 5% DMAHDM on the rate of Ca and PO_4_ is reported in Figure 4A,B. When comparing the 20% NACP formulation and 10% NACP formulation, an approximate three times increase in Ca and PO_4_ ions released was observed. However, as the NACP percentage load increased, the rate of diffusion of ions from the sealants increased approximately 5%. It appears that an increase in the NACP concentration by a factor of 1.5 does not provide the same corresponding increase in Ca and PO_4_ ion concentration over 70 days, which makes the 20%NACP formulations suitable for the proposed application.

Referring to previous studies from our group, this release concentration of Ca and PO_4_ ions was similar to release and recharge rates previously observed in a 30% NACP parental formulation with high inorganic filler content for reinforcement [17]. This variation could be attributed to the differences in the filler level in each formulation since in order for these ions to be available for remineralization, they must diffuse through the resin matrix, in this case, 50% PEHB.

The chemical composition of the formulation has a unique role for rechargeability. Since one of our objectives in this study was to develop ion-rechargeable resin-based sealants, PEHB resin matrix was chosen. The PEHB resin had shown high ion-recharging abilities in previous reports [19,22,23]. In the present study, the sealant showed promising ion recharging abilities because of the great amount of re-released Ca and PO_4_ ions after each cycle of ion-recharge. This is likely due to the acidic adhesive monomer PMGDM [18], which consists of a large part of the parental resin matrix PEHB. The suggested recharge mechanism of NACP is based on the ability of PMGDM chelate with the recharging Ca ions during the recharging process, and release the ions when it is exposed to the acidic environment, such as pH 4. Further study is needed to investigate Ca and P ion recharge and re-release mechanisms in solutions that mimic the oral environment, such as artificial saliva.

## 4. Materials and Methods

### 4.1. Development of Dental Resin Sealants

The resin matrix consisted of 44.5% of pyromellitic glycerol dimethacrylate (PMGDM) (Hampford, Stratford, CT, USA), 39.5% of ethoxylated bisphenol a dimethacrylate (EBPADMA) (Sigma-Aldrich, St. Louis, MO, USA), 10% of 2-hydroxyethyl methacrylate (HEMA) (Esstech, Essington, PA, USA), and 5% of bisphenol a glycidyl dimethacrylate (Esstech) [17]. 1% of phenylbis (2,4,6-trimethylbenzoyl)-phosphine oxide (BAPO) (Sigma-Aldrich) was added as a photo-initiator. This resin matrix is referred to as PEHB. To formulate a sealant with antibacterial properties, dimethylaminohexadecyl methacrylate (DMAHDM) was synthesized via a modified Menschutkin reaction using a method as described previously [22].

NACP was synthesized by a spray-drying technique according to previous methodology [21]. Briefly, calcium carbonate and dicalcium phosphate were dissolved in acetic acid to produce Ca ions with a concentration of 8 mmol/L and PO_4_ ions with a concentration of 5.3 mmol/L, then this solution was sprayed in a heated chamber using a spray-drying machine. An electrostatic precipitator was used to harvest the NACP with a particle size of average ± 116 nm. The silanized barium boroaluminosilicate glass particles were added for reinforcement purposes and had an average size of 1.4 µm (Caulk/Dentsply, Milford, DE, USA). The fillers were incorporated into the resin matrix at a filler level of 50%. Two main types of resin-based materials were available as pit and fissure sealants: filled flowable composite and unfilled resin-based sealants, both were used as commercial controls in this study. Hence, the following six sealants were tested.

1. Commercial Low-viscosity Sealant control termed “C-LV.”
  (FluroShield, Dentsply Caulk, Milford, DE, USA)”.
2. Commercial High-viscosity Sealant/Flowable Composite control termed “C-HV.”
  (Virtuoso, Den-Mat Holdings, Lompoc, CA, USA).
3. Experimental Sealant termed “5% DMAHDM + 0% NACP”
  (45% PEHB + 5% DMAHDM + 50% Glass + 0% NACP).
4. Experimental Sealant termed “5% DMAHDM + 10% NACP”
  (45% PEHB + 5% DMAHDM + 40% Glass + 10% NACP).
5. Experimental Sealant termed “5% DMAHDM + 20% NACP”
  (45% PEHB + 5% DMAHDM + 30% Glass + 20% NACP).
6. Experimental Sealant termed “5% DMAHDM + 30% NACP”
  (45% PEHB + 5% DMAHDM + 20% Glass + 30% NACP).

### 4.2. Flexural Strength and Flexural Modulus

Samples for flexural strength and flexural modulus testing were prepared using 2 × 2 × 25 mm stainless steel molds. Each paste was placed into the mold which was covered with Mylar strips and glass slides from both open sides of the mold then light-cured (500 mW/cm^2^, 60 s, Triad 2000, Dentsply, York, PA, USA) for on each open side. Samples were stored at 37 °C for 24 h. Flexural strength and flexural modulus were measured using three-point flexure with a 10 mm span at a crosshead-speed of 1 mm/min on a computer-controlled universal testing machine (MTS 5500R, Cary, NC, USA) [24].

Flexural strength (F) was calculated by using the following formula:

F = (3LS)/(2WH^2^), where L is the maximum load; S is the span; W is the width of the specimen and H is the height.

Flexural Modulus (M) was determined as:

M = (LS^3^3)/(4WH^3^3d), where L is the maximum load; S is the span; W is the width of the specimen, H is the height of the specimen, and d is the defluxion corresponding to the load L.

### 4.3. Flow Analysis

The recommendations of the ISO 6876/2012 and ANSI/ADA2000 standards were followed [25]. Briefly, two glass plates of 40 mm × 40 mm and approximately 5 mm thickness were used. The weight of one glass plate was approximately 20 g. The paste of each sealant was placed in the center of one of the glass plates using a graduated syringe. The amount of sealant was approximately 0.05 mL. The second glass plate was placed on top of the sealant; then a 100 g weight was used to make a total weight of approximately 120 g. After 10 minutes, the weight was removed, and the largest and smallest diameters of the discs formed by the compressed sealants were measured with the aid of a digital caliber (Mitutoyo MTI Corp., Huntersville, NC, USA) [26]. Six tests were done for each sealant.

### 4.4. Measurement of Initial Calcium and Phosphate Ions Release from NACP

Three specimens of approximately 2 × 2 × 12 mm were immersed in 50 mL of sodium chloride (NaCl) solution (133 mmol/L). The NaCl solution was previously buffered to pH 4 with 50 mmol/L of lactic acid to simulate a cariogenic low pH condition [27]. The specimen volume per solution ratio was almost 2.9 mm^3^/mL following previous study [22]. The tubes (*n* = 6) were kept in a 37 °C incubator during the experiment. Aliquots were collected at 1, 3, 5, 7, 9, 11, 14, 21, 28, 35, 42, 49, 56, 63 and 70 days. The Ca and PO_4_ ions concentrations from collected aliquots were analyzed using SpectraMax^®^ M Series Multi-Mode Microplate Reader from Molecular Devices [28]. The absorbance was measured using known standards and calibration curves. After each collection, the NaCl solution was replaced by a fresh solution. All the groups were tested for the initial Ca and PO_4_ ions release [29].

### 4.5. Calcium and Phosphate Ions Recharge and Re-Release

After the calcium and phosphate ions released for 70 days, the specimens were stored for almost 6 months before starting the ion recharging experiment to exhaust the ions in all the specimen and ensure there is no additional ion release. The pH of the immersion solutions was assessed by the same period of time. Then, these ion-exhausted specimens were used for the ion recharging experiment. The Ca ion recharging solution was made of 100 mmol/L CaCl_2_ and 50 mmol/L HEPES buffer [18]. The PO_4_ ion recharge solution was made of 60 mmol/L KHPO_4_ and 50 mmol/L HEPES buffer. Both solutions were adjusted to pH 7 by the use of 1 mol/L KOH [19]. Three specimens of approx. 2 × 2 × 12 mm were immersed in 5 mL of the calcium ion or phosphate ion recharging solution and gently vortexed for 1 min using Analog Vortex Mixer, (Fisher Scientific, Waltham, MA, USA) to simulate the action of using mouthwash [30]. Specimens were immersed three times for 1 minute each time. This is for a total of 3 min of ion recharge. After that, the recharged specimens were immersed in a 50 mL NaCl solution, which was adjusted to pH 4 as described in Section 4.4. This immersion was to measure the Ca and PO_4_ ion re-release on day 1, 2, 3, 5, 9, 11 and 14. After 14 days, the specimens were recharged again, and then the ion re-release was measured for 14 days at day 1, 2, 3, 5, 7, 9, 11 and 14 [31]. The same cycle or recharge and release were repeated for three times as described in Figure 1. The Ca and PO_4_ ion measurements were assessed in the same way as mentioned in Section 4.4.

### 4.6. Statistical Analysis

Kolmogorov-Smirnov test and Levene test were performed to confirm the normality and equal variance of data. The results of flexural strength, flexural modulus, flow and Ca and PO_4_ ion release and re-release were analyzed using one-way analysis of variance (ANOVA). Multiple comparisons between the different groups were conducted using Bonferroni’s multiple comparison tests. All the statistical analyses were performed by SPSS 22.0 software (SPSS, Chicago, IL, USA) at an alpha of 0.05.

## 5. Conclusions

The effects of different percentage loading of the remineralizing agent, NACP in new dental material formulations have been studied in a thorough and systematic approach. Considering the clinically relevant properties for dental sealants, the formulation containing 20% of NACP was selected as the optimal composition. The material flow was highly related to the mass fraction of the filler in the resin. At 20% NACP, the PEHB-based resin sealant provides high levels of Ca and PO_4_ ions release and durable repeated recharge capability, with no negative effect on the mechanical and physical properties of the sealant. Therefore, this is a promising approach to provide long-term ion release to promote remineralization and inhibit dental caries in occlusal surfaces of teeth in children and adolescents.
